# Career perspective: Charles M Tipton

**DOI:** 10.1186/s13728-015-0024-y

**Published:** 2015-04-17

**Authors:** Charles M Tipton

**Affiliations:** Department of Physiology, University of Arizona, Ina Gittings Building, Room 205 A, AZ 85721 Tucson, USA

## Abstract

This invited autobiographical article pertains to 52 years as an exercise physiologist of which 16 years were devoted to being an active emeriti. Although the career pathway was circuitous in nature, once resolved, it included preparation of future exercise physiologists; reducing the health hazards associated with the “making of weight” by scholastic wrestlers; using animals (rats and dogs) as the model system with a myriad of experimental procedure for obtaining insights and understandings of various exercise training mechanism in one-G environments, and in simulated μG environments. From the results, we have concluded that (a) inactivity, as represented by immobilization, is the most undesirable physiological state an animal should experience and (b) movement, as represented by training, will have an intrinsic adaptive influence on select biological tissues that, in some situations, can be independent of autonomic and hormonal influences.

## Introduction

I am extremely honored by the invitation to be included with the distinguished investigators that have proceeded me in presenting a *Career Perspective.* However, my pathway to becoming an experimental exercise physiologist was circuitous at best and lacked much of the serendipity experienced by Professor Peter Wagner [1]. Readers must realize that my primary involvement with extreme environments has been associated with 36 years of service on 11 different national committees devoted to evaluating research proposals concerned with the countermeasures necessary to offset the deleterious physiological consequences of microgravity with space travel and its exploration. These involvements culminated in the 2011 Decadal Study report to Congress [2,3]. A secondary role has been 12 years of NASA-supported research devoted to investigating the effects of simulated weightlessness [tail suspended, non-hindlimb weight bearing of head-down rodents] on physiological functioning. They resulted in 28 peer-reviewed publications and a 1995 NASA invitation from NASA Life Sciences Director Joan Vernikos to measure pre- and post-VO2 max values on rats flown in microgravity. Unfortunately, the measurement never occurred because of a last-minute change in the allocation of floor space within the spacecraft.

### The circuitous pathway

**Figure 1:**
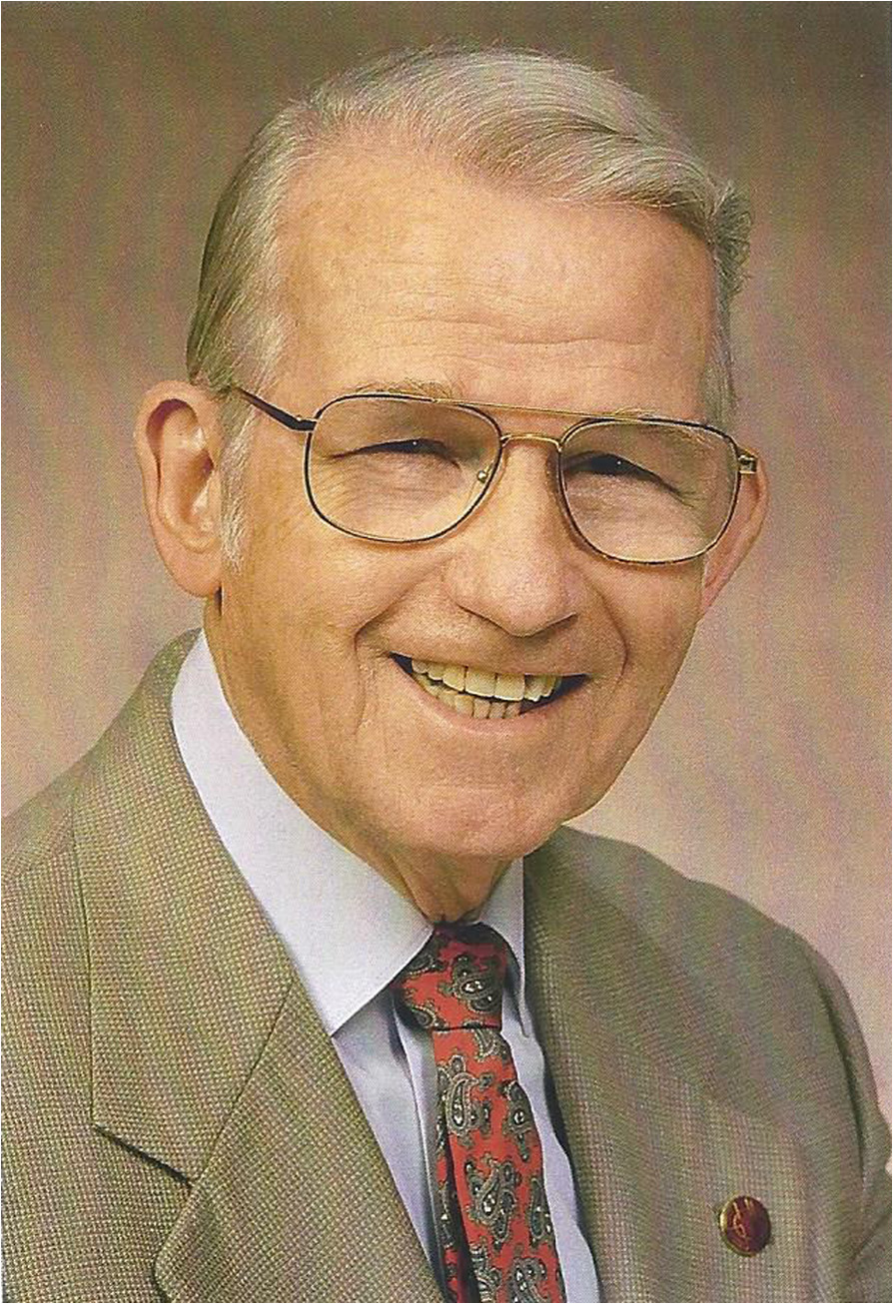
**Charles M Tipton at the University of Arizona.** From the collection of Charles M Tipton, photograph taken during the late 1990s.

On 29 November 1927, I was the third child born to Elizabeth White Tipton and Charles M. Tipton in Evanston, IL (Figure 1). My father was a Treasurer and senior accountant in a local furniture store in Winnetka, IL. During the early stages of *The Great Depression*, we moved to Dickerson, MD, which was a small town in a rural area located in the Northwest region of the state and approximately 35 miles from Washington, DC. Nearby were three uncles who were general, chicken and truck, or dairy farmers, respectively. Thus, a large percentage of my boyhood years was devoted to performing paid and unpaid chores on their farms as well as on our 10-acre property which contained animals (cows, chickens, rabbits) and a garden while lacking electricity and plumbing facilities.

The critical years of my high school education occurred during World War II, a time period when qualified science, mathematics, and physical education teachers were unavailable or reluctant to be located in remote rural areas while those individuals with expertise in agriculture were available and deferred. The end result was a vocational high school education with a major emphasis in agriculture and animal husbandry that had major deficiencies in mathematics plus the chemical and physical sciences. Another result during my senior year was being (a) selected to lead the daily calisthenics in the physical education period and (b) designated to organize class indoor-outdoor sporting events.

Because of deficiencies in visual acuity, I was rejected for duty by the Armed Services during the war years; however, after becoming 18 years of age, I was accepted by the Army and served 2 years in the Army of Occupation in Japan (1946–1948). The Army sent me to “school” to become a physical fitness instructor, a duty I performed not only for stateside basic training camps but also for an infantry company in Japan (25th Division). After discharge (1948), I became a Physical Education Major at the University of Maryland where I remained for 2 years before transferring to Springfield College in MA. Two years later, I graduated with a BS in Physical Education (1952). Subsequently, I obtained a Teaching Assistantship at the University of Illinois and completed an MS dissertation in Physical Education under the supervision of Professor Thomas K. Cureton, Director of their Physical Fitness Laboratory.

Unexpectedly, after 2 years of teaching and coaching in the high schools in rural regions of Illinois, I found the experience was not rewarding, challenging, or satisfying. Hence, the decision was made to pursue a Ph.D. degree. Later, I accepted an Assistantship in Health Education with the responsibility to provide exercise therapy for disabled students at the University of Illinois with Professor Howard Hoyman as my advisor. The degree had prerequisites necessary for acceptance into medical schools; thus, the early years at the university were devoted to becoming a undergraduate science major while securing the necessary advanced courses for graduation purposes. To meet the needs of a growing family, it was necessary to supplement my income with part-time employment. Fortunately for financial and personal reasons, I became acquainted with Professor Darrell M. Hall of the College of Agriculture who had research responsibilities associated with the Extension Service that was funded by the United States Department of Agriculture.

One of Professor Hall’s main duties was to direct the research activities of the Extension Service which involved the health projects of Illinois 4-H Clubs. They included physical fitness testing in most of Illinois 101 counties. Therefore, my summer responsibilities for the next 5 years required me to lead a testing team to conduct one or more 4-H Physical Fitness sessions/day, explain the results to participants and parents, and to perform a statistical analysis of the results [4]. It was this experience, under his tutelage, that stimulated my interest in conducting physiological research and to seek scientific explanations for the results. After completing a 1-year theory and laboratory course in Mammalian Physiology directed by Professor Frederick R. Steggedra and a semester of a theory and laboratory course in Comparative Physiology directed by Professor C. Ladd Prosser, I became passionate about a future in physiology and, with Professor Hall’s encouragement, requested a transfer to the Department of Physiology with Professor Steggedra as my mentor. Happily, it was approved.

Professor Steggedra was an outstanding advisor who was supportive and caring in all phases of the degree process. Moreover, he instilled in me a great love for surgical research. However, the Illinois faculty member who inspired me the most by his intelligence, integrity, and comprehension of science and physiology was Dr. Robert E. Johnson, formerly from the Harvard Fatigue Laboratory [5].

After completing my dissertation research on the mechanisms of the bradycardia of training in rats, I accepted the opportunity in 1961 to become an Assistant Professor of Research at Springfield College with the responsibilities of teaching the theory and laboratory courses in Exercise Physiology and conducting the research projects of grants awarded to Professor Peter V. Karpovich. In essence, it was a post-doctoral experience before such appointments became expected for graduating physiologists. From this experience, important insights were gained on how to conduct laboratory research while acquiring an admiration of Professor Karpovich’s ability to evaluate and analyze experimental data. Two years later, a joint appointment offer from the University of Iowa to become an Assistant Professor in the Department of Physical Education-Men, College of Liberal Arts, and in the Department of Physiology and Biophysics, College of Medicine, was accepted with the funding provided by the College of Liberal Arts.

### Career academic and research contributions

#### Academic contributions

**Figure 2:**
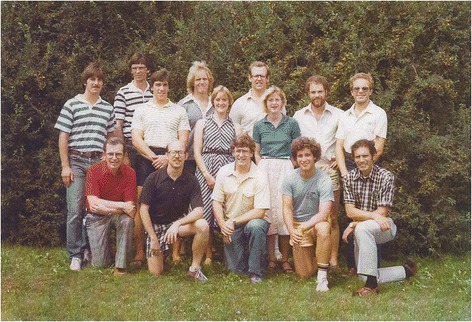
**Select individuals associated with the laboratories of Charles M. Tipton and Carl V. Gisolif at the University of Iowa.** Except W. Mitchell, all were enrolled in the Ph.D. program that emphasized Exercise Physiology. Photograph taken in late 1970s. Back row, left to right: K. Marcus, L. Louters, T. Wall, W. Mitchell, J. Edwards, M. Owens. Middle row, left to right: M. Sturek, J. Fruth, P. Kershner. Front row, left to right: C. Tipton, R. Oppliger, T. Bedford, M. Overton, C. Gisolfi. From the collection of Charles M. Tipton.

At the time of the 1963 appointment, Physical Education Departments within the Big Ten Conference had agreed to revise their Ph.D. degrees into a discipline-oriented specialization program which emphasized anatomy and histology, biomechanics, exercise physiology, motor control or motor learning, sports history, and administration [6]. Thus, my first task at Iowa was to develop a graduate degree program in exercise physiology. It was one whose prerequisites included completion of undergraduate courses in the mathematical, biological, physical, and chemical sciences necessary to enter medical school. In addition, students were expected to successfully complete theory and laboratory courses in medical physiology and biochemistry plus classes in cardiovascular pharmacology and endocrinology that were offered either by the Colleges of Medicine or Liberal Arts. Requirements also included graduate courses in biomechanics, motor learning, mastering one foreign language, statistics with research methodology, dissertation research plus theory, and laboratory courses in exercise physiology. With strong support from the Chair, Dr. Louis E. Alley, the program was implemented in 1964, added Dr. Carl V. Gisolfi as the Assistant Director in 1970 and supported by the Graduate College, National Defense Education Act plus National Institute of Health training grant and renewal funds. Interestingly, the renewal application was criticized by reviewers because the “high standards” were causing an elevated drop-out rate. By 1986, the program had graduated 26 individuals with Ph.D. degrees of which 25 (one graduate subsequently developed his own company) had secured academic appointments in select universities throughout the United States (Figure 2). Recently, the publication records of the graduates were evaluated for presentation at a 2015 symposium and according to a 3-25-2015 communication from Dr. Kenneth Baldwin, their collective productivity was approximately 2,000 publications.

Like other universities, after that date, the Physical Education—Men’s Department at the University of Iowa changed its name to Exercise Science and had modified the Ph.D. requirements for specialization degrees. However, two potential national contributions emerged from the establishment of a program emphasizing exercise physiology; namely, helping to change the perception that (a) graduate programs in Physical Education were able to produce qualified individuals to conduct exercise physiology research and (b) enhancing the names changes of Departments of Physical Education to Departments of Movement Science, Kinesiology, Exercise Science or Exercise and Sport Sciences or combinations thereof.

### Human research contributions

Readers must understand that citizens of Iowa have two strong passions; specifically, the economic benefits of growing corn and the achievements of their local scholastic and collegiate wrestling teams. Thus in 1967 when physicians from Muscatine County recommend the abolishment of scholastic wrestling because local high school wrestlers were “making certification wrestling weights” by procedures that were hazardous to their health [7], the pronouncement created a turmoil for the Iowa High School Athletic Association (IHSAA) and for the Sports Medicine Committee of the Iowa Medical Society (IMS). Since Professor Hall had developed an anthropometrical method to predict a physically fit body weight for 4-H Club members from a database of more than 30,000 subjects between 10 and 18 years of age [4], I proposed the use of the Hall Method to assess a minimal wrestling weight (MWW) for scholastic wrestlers in Iowa. After measurements were made from finalists at the 1968 Iowa state championship and from wrestlers from Iowa City high schools, we found that a high correlation coefficient agreement existed between the actual and predicted body weights [7], Table 1. However, when analyzed by specific weight classes, the method was effective only for individuals weighing less than 138 lb (62.73 kg) and vastly ineffective for predictive purposes for wrestlers weighing more than 155 lb (70.45 kg.): presumably, because the Iowa males were older and more physically mature than the 4-H club members [7]. In essence, it was a bitter but an important lesson to learn. Specifically, we need to have an available definitive population database before making speculations. Although the recommendation made to the IHSAA and to the IMS was not to adopt the Hall Method for determining a MWW, we remained convinced that with a scholastic wrestler database, it would be possible to scientifically predict a MWW for Iowa high school students well in advance of the competitive season. Thus in 1968, the Iowa Wrestling Study (IWS) began with the naive aspiration of having a MWW program being implemented by 1978, and hopefully, in other states within one or two decades. It was our contention that a MWW should be no less than 5% body fat [8].

**Table 1:** **Evaluation of the Hall Method to predict the minimal wrestling weights of non-finalists and finalist wrestlers (1968–1969)**

**Number**	**Actual body weight (lb)**	**Predicted Hall body weight (lb)**	**Correlation coefficient**	**Reference**
282	133 ± 1.7	133 ± 1.4	0.95	[7]

Values are means and standard deviations.

The realities obtained in a protracted manner and detailed in Table 2 demonstrated that it required 25 years for Wisconsin to become the first to implement a statewide MWW program [9]. Moreover, it took 38 years before the state of Iowa adopted a MWW program and it occurred only after (a) the occurrence of three deaths by collegiate wrestlers in 1997 who had followed hazardous health practices [10], (b) forceful action by the Center for Disease Control (CDC) on the National Collegiate Athletic Association (NCAA) to modify existing weight allowances [11] and (c), and a 9 year delay before the National Federation of High School Associations mandated states to implement weight loss procedures that prevented scholastic wrestlers from losing more that 1.5% body weight per week while maintaining no less than 7% body fat for males and 12% for females ([12], p.8). As emphasized by Casperson (a CDC employee), the existence of a regulated national-wide MWW program for an estimated 270,000 scholastic wrestlers will markedly prevent and discourage students from utilizing or following undesireable health practices to make weight [11]. While it is satisfying to know our MWW concept was ultimately implemented on a national scale, it is not rewarding to realize that it was driven more by the tragic deaths of three individuals than by the logic of scientific merit (Table 2).

**Table 2:** **The historical record associated with the search for a minimal wrestling weight (MWW) for scholastic wrestlers**

**Historical record**			
1.	These investigations include The Iowa Wrestling Study (IWS: 1968–1988), the Midwestern Wrestling Study (MWS: 1986–1991) and the Wisconsin Wrestling Minimal Weight Project (WPWMWP: 1989–1998)		
2.	The *IWS* includes certification results from 8,900 students, questionnaire findings from 582 students, body measurements from 2,536 subjects, and visitations to approximately 55 high schools. Salient findings were [39-42].		
	a.	Approximately 40% of the students receive certification to wrestle in weight classes between 119–139 lb (54.06–63.19 kg) whereas 57% of the students become certified to wrestle in weight classes 112–145 lb (50.90–65.90 kg). Consequently, the weight class system creates conditions favoring undesirable practices. A recommendation to have matches with more than one individual per weight class was ignored	
	b.	The majority of wrestlers believe or practice the following [39,43].	
		(1)	Performance will not change because of losing weight.
		(2)	Other wrestlers and the coach should be consulted on how to make weight
		(3)	Local physicians will seldom or never be consulted on how to make weight
		(4)	If there is more than 9% of one’s body weight to lose, it is acceptable to use a rubber suit and to exercise in the heat.
	c.	We have learned the following:	
		(1)	Scholastic wrestlers do not lose weight in a systematic manner, most of it is lost in the final days of certification [39]
		(2)	The individuals who lose the highest percentage of their body weight are the youngest and located in the lower weight classes [39,41]. Of 747 wrestlers, 8% will lose 10% of their body weight in a few days, and one or two will lose 20% or more in the same time period [39]
		(3)	Urinalysis finding indicate finalists are dehydrated before and during the competition as demonstrated by elevated values for specific gravity, osmolarity, potassium, proteins and ketones. The data also showed glomerular filtration rates were reduced [44]
		(4)	Although we have recommended a MWW be one with no less than 5% fat [45], we found that 33% of the contestants (*N* = 47) had fat percentages lower than this value. All were in weight classes lower than 131 lb (54.94 kg) and all were among the youngest of the competitors [45]
3.	Since the IMS or IHSAA was unresponsive to our report and its recommendations [46], we (Tipton, Oppliger, Tcheng) organized the MWS and combined forces with investigators from the states of Illinois, Minnesota, Nebraska, and Ohio. They were interested in the MWW concept and were active with their respective state associations to implement such a program. Select results were the following [47]:		
	a.	Developed a scholastic wrestler data base from 860 individuals that included stature, body diameters (*n* − 7), body circumferences (*N* = 10), body skinfolds (*N* = 9), body density, and fat-free mass (FFB).	
	b.	Used the statistical expertise of Thorland, Lohman, and Tcheng to perform cross validations of 16 different equations plus 9 new ones. We found that the skinfold equation of Lohman [48] was the equation of choice because it had the lowest constant and total errors [47].	
4.	The availability of a practical MWW equation had an impact on an individual (Herrmann) associated with the Wisconsin Interscholastic Athletic Association (WIAA) and on two physicians associated with the University of Wisconsin (Harms and Landry), all of whom were deeply concerned about the problems of “making weight.” They invited Dr. Oppliger from the University of Iowa to join them and collectively initiated the Wisconsin Wrestling Minimal Weight Project (WWMWP [9]).		
	a.	WWMWP advocated a 3-year trial period beginning in 1989 using the Lohman equation for a minimum wrestling weight of 7% fat that allowed no more than a loss of 3 lb (1.36 kg) per week which had the full support of Wisconsin Dietetic Association and the Wisconsin Department of Public Instruction. However, the Wisconsin Wrestling Coaches Association was not supportive of the project [49]	
	b.	In 1991, WIAA mandated all high schools with wrestling programs follow the procedures established by the WWMWP. Besides having the support of parents, wrestlers, administrators, and various associations, it also became accepted by the coaches. It is of interest that by 1994, there was a 6% increase in the number of individuals who became certified [49].	
	c.	These developments had minimum impact on Iowa officials or on members of the Wrestling Rules Committee of the National Federation of State High School Associations (NFSHSA)	
5.	In 1997, three collegiate wrestlers died in their attempts to “make weight” [10]. According to Casperson [11], an employee of the Center for Disease Control (CDC), it was the CDC that “encouraged” the National Collegiate Athletic Association (NCAA) to instantly modify the rules for the making of weight by collegiate wrestlers		
6.	In 2005, the NFSHSA mandated that beginning with the 2006–2007 competitive season, all states that implemented a program pertaining to a minimal wrestling weight for high school students that included a body weight that has no less than 7% fat for males and 12% for females would allow no more than a 1.5% loss in body weight per week, while permitting finalists to gain 1 lb (0.45 kg) per day during tournament competition [12].		

## Animal research contributions and their insights

### Overview

The goal of our Exercise Physiology Laboratory was to conduct acute and chronic exercise studies with experimental animals to better understand responsible mechanisms. Table 3 provides a profile of the species utilized and their inclusion within various studies. We have been pleased to have been associated with select tests and equipment necessary to determine the trained state of animals that has been utilized by other investigators [13,14]. However, it is likely that our most important contribution to animal research was joining Dr. Phillip Gollnick from the Department of Physical Education at Washington State University in (a) defying the unofficial mandate of Professors Thomas Cureton and Peter V. Karpovich that animal research should not be conducted in Departments of Physical Education within the United States [15] and (b) by resisting the collective rejection of 13 animal abstracts for presentation at the 1970 meeting of the American College of Sports Medicine (ACSM) that were denied publication in *Medicine and Science in Sports* for the same reasons. However, since 1971, no abstract has been rejected by ACSM for presentation at their annual meetings or for publication in *Medicine in Science and Sports* or in *Medicine and Science in Sports and Exercise* solely because it pertained to animals.

**Table 3:** **A profile of the animal research activities of Charles M. Tipton (1960–1998)**

**Animal research activities**				
A.	Experimental animals			
	1.	Rodents (rats, both sexes and all studies (*N* = approximately 1,900)		
	2.	Mongrel dogs (males only, cardiovascular and connective tissue studies (*N* = approximately 80)		
	3.	Non-human primates (*Galgo senegalenis*, both sexes, systolic blood pressure and connective tissue studies (*N* = approximately 25)		
B.	Experimental animal studies			
	1.	Development of exercise protocols and tests for dogs and rats		
	2.	Investigations on the bradycardia of training		
		a.	Normal	
		b.	Diencephalon lesioned	
		c.	Right and left unilateral vagectomized	
		d.	Immunosympathectomized (IS)	
		e.	Thyroidectomized	
		f.	Adrenalectomized	
		g.	Hypophysectomized	
		h.	Isolated hearts (Langendorff)	
	3.	Investigations on the influence of chronic exercise on systolic blood pressure		
		a.	Normotensive, non-human primates	
		b.	Normotensive , rats	
		c.	Normotensive and aging (2 years)	
		d.	Normotensive and a high fat diet	
		e.	Normotensive and injections of desoxycortosterone acetate (DOCA)	
		f.	Hypertensive because constriction of the renal artery	
		g	Dahl salt sensitive hypertensive rats	
		h	Dahl salt resistant hypertensive rats	
		i	Spontaneously hypertensive rats (SHR)	
		j	SHR and high calcium diets	
		k	SHR and low calcium diets	
		l	SHR and IS	
		m	SHR and adrenal demedullation (DM)	
		n	SHR, IS, and DM	
		o	SHR and Strokeprone (SHR-SP)	
		p	SHR-SP and static exercise	
		q	SHR and post-exercise hypotension	
		r	Hypophysectomizied (Hyphx)	
	4.	Investigations on the influence of acute and chronic exercise on the oxygen transport system		
		a.	Normal	
		b.	SHR	
		c.	Hyphx	
	5.	Investigations on the influence of inactivity (immobilization) plus acute and chronic exercise on ligaments		
		a.	Normal	
			(1)	Aging
			(2)	Surgical repair
		b.	Hormonally deprived [Hyphx and thyroidectomized (Thyrx)]	
			(1)	Surgical repair.
			(2)	Hyphx and replacement hormones [adrenal corticorticotrophin (ACTH), growth hormone (GH), interstitial-cell-stimulating hormone (ICSH), and thyroid-stimulating hormone (TSH).
	6.	Investigations on the influence of simulated microgravity on select physiological systems		
		a.	Normal with both hindlimbs non-weight bearing	
		b.	Normal with a single hindlimb non-weight bearing	
		c	Hyphx with both hindlimbs non-weight bearing	

### Insights: exercise and training

Using immobilization as an extreme example and non-weight bearing by hindlimbs via tail suspension as a moderate example of inactivity, it is apparent that lack of physical activity or exercise has a deleterious physiological effect. However, when the effects of chronic exercise are evaluated by measurements pertaining to reductions in exercise and resting heart rates; lower heart rates after atropine injections; lower exercise and resting systolic or mean blood pressures; increased tissue mass; decreased adipocyte dimensions; elevated tissue strength; higher levels of cytochrome oxidase activity; changes in tissue transmitter concentrations; improved run times; and enhancement of the work performed and in augmented VO_2_ max values, the results demonstrate that trained rats will exhibit changes at select time periods throughout the experiment (Table 3). Furthermore, we suggest that the process of training in animals will have an intrinsic adaptive influence on select bodily tissues that can occur in animals from the various experiment groups that indicates, in some circumstances, an independence from autonomic and hormonal influences (Table 3).

### Insights: exercise and resting bradycardia

Despite its long history of investigation, the explanation for the bradycardia in training remains unresolved. In fact, it has been the “the big question” that our laboratory has been seeking to answer since 1960. The main insight from our animal studies is that it can occur in the eight different experimental groups [16] listed in Table 3. Even though it was not present in the adrenalectomized animals at every time period, lower heart rates were observed after 30–40 days of training; after injections of atropine; and during a 9-min sub-maximal exercise test [17]. We were extremely surprised to learn that lower heart rates were not a characteristic of the isolated hearts associated with a Langendorff preparation [18] as we felt it was an internal adaptation. While we support the concept that cholinergic influences are increased by training whereas sympathetic influences are decreased with chronic exercise [16], we cannot ignore the data favoring intrinsic changes in the SA node [19]. Even though our laboratory has failed to identify the responsible mechanisms, we continue to speculate that a combination of non-neural acetylcholine and the presence of latent pacemakers are contributing to the cholinergic effect.

### Insights: exercise and blood pressure

Results from normotensive animals leave little doubt that training will markedly lower resting and acute exercise blood pressures [20,21]. However, it has been our experience when training genetic models of hypertension; (a) training will never normalize resting or exercise pressures and (b), training animals in excess of 40%–60% VO_2_ max will result in elevated, not lower, pressures [22]. Of the ten hypertensive groups that were trained by dynamic aerobic exercise, five failed to exhibit lower resting blood pressures. Two were pertained to the kidney (renal constriction and Dahl SS rats) [23], one to consuming a high Ca++ diet, one to being SHR-SP, and one to being IS (sympathectomized) and DM (adrenal demedullated) [24]. Frankly, we were surprised by these negative results and have no meaningful explanation for them. Since SHR-IS rats have exhibited lower resting pressures with training, we suggest that the adrenal medulla is necessary for a training effect to occur in these experimental animals [24]. Because SHR-SP animals were unresponsive to dynamic aerobic exercise, we investigated whether static (isometric) exercise would increase the incidence of strokes as demonstrated by histological analysis [25]. Despite media statements and the fact that MBPs approached 290 mmHg, we were unable to prove that static exercise per se would elicit strokes [26]. Furthermore, we have learned that deaths of SHR-SP populations were not always from the result of strokes [26]. With acute exercise, we found that SHR groups will exhibit evidence for post-exercise hypotension [27], a finding that is among the first for animals but known to occur in humans for over a century [28]. To explain resting reductions in systolic blood pressure with aerobic exercise, we favor the resetting of baroreceptors, reductions in sympathetic tone, increased lumen area, and decreased total peripheral resistance [20-22].

### Insights: chronic exercise and the oxygen transport system (VO_2_max)

Because of ineptness during the early years, our laboratory was unable to perfect a suitable chamber to measure the oxygen consumption of exercising dogs. However, this changed in 1979 with the availability of a suitable chamber for assessing exercise performance and work accomplished or for training status of rats [14]. In essence, the “gold standard for rats has arrived.” Since that date, every experimental rat group listed in Table 3 has been measured before, during, and after the experimental period to determine whether a trained state existed. As for responsible mechanism, we endorse the perspective advanced by Snell, Levine, and Mittchel in that it is the result of factors that insure maximal heart rates, stroke volumes, cardiac outputs, and maximal systemic a-v O_2_ differences [29]. Our laboratory takes great pride in being among the first to demonstrate trained hypophysectomized rats have significantly higher VO_2_ max values than their non-trained controls [30]. One aspect is certain; training can result in select adaptations in the absence of hormones from the anterior pituitary gland.

### Insights: chronic exercise and its influence on ligaments

In the 1960s, a controversy prevailed concerning the effects of exercise on ligaments in animals [31]. Consequently, our laboratory, with the assistance of the College of Medicine Machine Shop, developed the equipment to measure the strength of ligaments [31,32]; specifically, the medical collateral ligament of the knee joint. Strength measures were identified as either separation force or junction strength because intact ligaments separate from the bone at the site between non-mineralized and mineralized fibrocartilage [33]. In the spectrum of activity, we have evidence that inactivity is associated with weaker ligaments and training with significantly stronger ones [31,33]. In addition, the inactivity caused by protracted immobilization is absolutely the worst physiological state for intact and especially for repaired ligaments. In fact, we have demonstrated in dogs that a repaired ligament that was not immobilized was significantly stronger than those that were immobilized [33]; presumably, because the dog was judicious in using minimal weight bearing by the experimental leg during the 6-week recovery period. Training by dogs will gradually increase the strength of repaired ligaments but is unable to achieve normalization in 12 weeks and unlikely in 26 weeks [33]. Similar results were obtained with hypophysectomized rats that had either intact or repaired ligaments [33,34]. When replacement hormones were administered, testosterone, ICSH, and TSH enhanced the strength of intact ligaments whereas TSH had no impact on repaired ligaments. Besides immobilization, ACTH, TSH, and thyroxine administration were associated with weaker ligaments [33]. For repaired ligaments, training plus testosterone and ICSH will enhance strength whereas immobilization plus ACTH, TSH, and thyroxine were identified with lower strength values [33]. We believe that training and immobilization are affecting collagen metabolism at the junction between non-mineralized and mineralized fibrocartilage as well as at the repair site. However, we failed to determine whether their influence was affecting the equilibrium relationship between synthesis and degradation [35].

### Insights: simulated weightlessness and exercise performance (VO_2_ max)

Although the ultimate goal was to measure the VO_2_ max of rats before, during, and after exposure to conditions of microgravity (μG), we were pleased to be considered for before and after measures. When that opportunity did not occur, our laboratory conducted simulated weightlessness studies with tail-suspended rats. When humans are exposed to sustained periods of μG, VO_2_ max and exercise performance will decrease [3]. When both non-trained (NT) and trained (T) rats were suspended, the T exhibited greater reductions than the NT; but both groups exhibited significantly lower values [36]. Moreover, both groups showed significantly slower run times and marked reductions in the mechanical efficiency of running [36], changes due, in part, to the atrophy experienced by the skeletal muscles. Interestingly, suspension of Hyphx animals had no significant effect on VO_2_ max values even though significant atrophy occurred in the soleus and plantaris muscles and they exhibited significantly slower run times [37].

To assess the importance of weight bearing on tissue mass, we designed a suspension apparatus that allowed for single-leg weight bearing (20% of body mass) [38]. At the end of 14 days, weight bearing prevented the loss of mass in the soleus, plantaris, and gastrocnemius muscles and maintained iliac blood flow but was unable to retain citrate synthesis activity. On the other hand, the freely hanging leg exhibited significant loss of muscle mass and reductions in iliac blood flow and in the activity of aerobic enzymes [38].

### Residues

Like millions of others, I was affected by the conditions of World War II and would not wish to experience those conditions again. My major regret from that era was not being convinced of the importance of an education that emphasized rigorous courses in mathematics plus the biological, chemical, and physical sciences. Besides my graduate students, the individuals whom I have admired and have influenced me the most (in alphabetical order) have been Professors, Jerry A. Dempsey, V. Reggie Edgerton, Philip D. Gollnick, Allan R. Hargens, John O. Holloszy, and Carl V. Gisolfi. Of the insights, none have acquired the significance of the 1970 formation of the Iowa-Washington State Alumni and Friends Group that added Arizona after 1984. In 2015, the 45th meeting of this group will be held at the ACSM Convention in San Diego. This meeting is significant, because to me, personal relationships are far more important than scientific findings.

Thank you for allowing me to present a perspective on my career.
